# Neutralizing Antibody Induction Associated with a Germline Immunoglobulin Gene Polymorphism in Neutralization-Resistant SIVsmE543-3 Infection

**DOI:** 10.3390/v13061181

**Published:** 2021-06-21

**Authors:** Yuto Nomura, Saori Matsuoka, Midori Okazaki, Takeo Kuwata, Tetsuro Matano, Hiroshi Ishii

**Affiliations:** 1 AIDS Research Center, National Institute of Infectious Diseases, Tokyo 162-8640, Japan; yuzujin@niid.go.jp (Y.N.); s-matsu@nih.go.jp (S.M.); omidori@niid.go.jp (M.O.); tmatano@nih.go.jp (T.M.); 2 Department of Computational Biology and Medical Sciences, Graduate School of Frontier Sciences, University of Tokyo, Tokyo 108-8639, Japan; 3 Division of Clinical Retrovirology Joint Research Center for Human Retrovirus Infection, Kumamoto University, Kumamoto 860-0811, Japan; tkuwata@kumamoto-u.ac.jp; 4 Division of Vaccine, Joint Research Center for Human Retrovirus Infection, Kumamoto University, Kumamoto 860-0811, Japan; 5 Institute of Medical Science, Graduate School of Medicine, University of Tokyo, Tokyo 108-8639, Japan

**Keywords:** SIV, neutralizing antibody, B cell receptor, germline, polymorphism

## Abstract

Antibody responses are crucial for the control of virus infection. Understanding of the mechanism of antibody induction is important for the development of a vaccine eliciting effective anti-virus antibodies. Virus-specific B cell receptor (BCR)/antibody repertoires are different among individuals, but determinants for this difference remain largely unclear. We have recently reported that a germline BCR immunoglobulin (IgG) gene polymorphism (VH3.33_ET or VH3.33_VI) in rhesus macaques is the determinant for induction of potent B404-class anti-simian immunodeficiency virus (SIV) neutralizing antibodies in neutralization-sensitive SIVsmH635FC infection. In the present study, we examined whether neutralization-resistant SIVsmE543-3 infection can induce the anti-SIV neutralizing antibodies associated with the germline VH3.33 polymorphism. Anti-SIVsmE543-3 neutralizing antibodies were induced in all the macaques possessing the VH3.33_ET allele, but not in those without VH3.33_ET, in the chronic phase of SIVsmE543-3 infection. Next generation sequencing analysis of BCR VH genes found B404-class antibody sequences only in those with VH3.33_ET. These results indicate that anti-SIVsmE543-3 neutralizing antibody induction associated with the germline BCR IgG gene polymorphism can be triggered by infection with neutralization-resistant SIVsmE543-3. This animal model would be useful for the elucidation of the mechanism of potent antibody induction against neutralization-resistant viruses.

## 1. Introduction

Development of an effective vaccine is a key for global control of infectious diseases. An antibody is a major acquired immune effector to prevent and/or control virus infection. Efficiency of antibody induction by vaccination is affected by both vaccine-related and host-related factors [1,2]. Regarding the latter, multiple factors, including host immune conditions and genetic factors, are considered to have an influence on immunogenicity, however most of them have not fully been elucidated [3,4,5]. Determination of host-related factors associated with potent antibody responses would contribute to our understanding of the mechanism for efficient antibody induction.

Potent antibodies are produced from plasma cells differentiated from mature B cells with higher antigen-B cell receptor (BCR) binding affinity [6,7]. Maturation of B cells with somatic mutations in BCR genes is induced by repeated antigen-BCR interaction following the initial priming of naïve B cells [8,9]. BCR genes in individual B cells are reconstituted by VDJ recombinations of germline immunoglobulin (Ig) genes and determine antigen specificity of B cells. Recent studies have reported polymorphisms in Ig-heavy chain variable genes, suggesting a possible effect of the polymorphisms on antibody induction [10,11].

We previously reported a potent monoclonal anti-simian immunodeficiency virus (SIV) B404, and related B404-class neutralizing antibodies (NAbs), induced in rhesus macaques infected with SIVsmH635FC, a NAb-sensitive SIV strain obtained by passage from NAb-resistant SIVsmE543-3-infected macaques [12,13]. The B404-class NAbs, consisting of heavy chains having a variable region (VH), VH3.33 [14], with long complementarity determining region 3 (CDR3), and λ light chains, recognize a conformational epitope comprising SIV Env V3 and V4 loops, and have potent NAb activity against various SIV strains [15]. Recently, we have found a polymorphism in the germline Ig VH3.33 gene (VH3.33_ET (38E-65T) or VH3.33_VI (38V-65I)), which is associated with B404-class NAb induction in SIVsmH635FC infection [16]. Infection of macaques possessing the VH3.33_ET allele with NAb-sensitive SIVsmH635FC induced B404-class antibodies that neutralize not only NAb-sensitive SIV strains, but also NAb-resistant SIVsmE543-3.

In the present study, we examined the effect of the germline Ig VH3.33 polymorphism on antibody induction in neutralization-resistant SIVsmE543-3 infection. Anti-SIVsmE543-3 NAb responses were induced only in rhesus macaques possessing the VH3.33_ET allele. Next-generation sequencing (NGS) analysis of BCR VH genes detected B404-class antibody sequences only in those macaques with VH3.33_ET. These results indicate that NAb induction associated with the germline Ig VH3.33 polymorphism can occur in NAb-resistant SIVsmE543-3 infection.

## 2. Materials and Methods

### 2.1. Animal Experiments

This study was performed using frozen samples obtained in our previous study. In that study, seven Burmese rhesus macaques were intravenously infected with 100 TCID_50_ (50% tissue culture infective dose) of SIVsmE543-3. Viruses were obtained from COS-1 cells transfected with the molecular clone SIVsmE543-3 DNA (Genbank accession number U72748) [17] and propagated on rhesus macaque PBMCs to prepare the SIVsmE543-3 inoculum stock. Four (#4, #5, #6, and #7) of the seven macaques received a vaccine consisting of a DNA and a Sendai virus (SeV) vector expressing SIVmac239 Gag [18,19] approximately 3 months before the SIVsmE543-3 challenge. Data on viral loads and T-cell responses in macaques #6 and #7 were previously reported [19], but data on the remaining five macaques have not been published. The previous animal experiments were carried out in the Tsukuba Primate Research Center (TPC), National Institutes of Biomedical Innovation, Health and Nutrition (NIBIOHN, Tsukuba, Japan) with the help of the Corporation for Production and Research of Laboratory Primates (Tsukuba, Japan) after approval by the Committee on the Ethics of Animal Experiments in NIBIOHN and the National Institute of Infectious Diseases, under the guidelines for animal experiments and in accordance with the Guidelines for Proper Conduct of Animal Experiments established by Science Council of Japan [20]. Blood collection, vaccination, and virus inoculation were performed under ketamine anesthesia.

### 2.2. Determination of Germline VH3.33 and TRIM5 Alleles

Germline VH3.33 polymorphisms were determined by direct sequencing of PCR amplified VH3.33 genes as described before [16]. VH3.33 genes were amplified by Premix Taq (TAKARA BIO, Kusatsu, Japan) using VH3.33-specific primers, VH333F (5′-TCTCTTGTGTAGCCTCTGGGT-3′) and VH333R (5′-GCTGTTCATTTGCAGAAACAGTGA-3′). VH3.33 allele was determined by direct sequencing of PCR products using dye terminator chemistry and an ABI 3500xL genetic analyzer (Applied Biosystems, Thermo Fisher Scientific, Tokyo, Japan). Alternatively, the PCR products were subcloned into TOPO TA Cloning vector (Invitrogen, Thermo Fisher Scientific, Tokyo, Japan) and sequenced.

TRIM5 polymorphisms were determined as described previously [21]. Briefly, peripheral blood mononuclear cells (PBMCs)-derived genomic DNAs were subjected to PCR amplification of a region between TRIM5 exon eight and 3′ UTR, using 5′-TGACTCTGTGCTCACCAAGCTCTTG-3′ and 5′-ACCCTACTATGCAATAAAACAT TAG-3′ primers to determine TRIM5α or TRIMCyp. A TRIM5 exon eight region was amplified by PCR using 5′-CTTCTGAACAAGTTTCCTCCCAG-3′ and 5′-ATGAGATGC ACATGGACAAGAGG-3′ primers and subjected to direct sequencing to determine TRIM5α_TFP or TRIM5α_Q.

### 2.3. Analysis of Plasma Neutralizing Activity

The neutralizing activity of plasma samples was measured by examining the reduction in infection-induced luciferase activity in TZM-bl cells by addition of plasma as described before [15,16]. SIVs were obtained from 293T cells transfected with the molecular clone DNAs of SIVmac316 [22], SIVsmH635FC [12], and SIVsmE543-3 [17], respectively. Serially diluted samples in duplicate were incubated with 200 TCID_50_ of SIVs (SIVmac316, SIVsmH635FC, or SIVsmE543-3) at 37 °C for 1 h and added into 1 × 10^4^ TZM-bl cells in a 96-well plate. Two days later (or three days later for anti-SIVsmH635FC NAb assay), cells were lysed, and the luciferase activity was measured using Beta-Glo assay system (Promega, Tokyo, Japan).

### 2.4. NGS Analysis of BCR IgG VH cDNAs

NGS analysis of BCR IgG VH3 cDNAs was performed as described previously [16]. Total RNAs were extracted from 5 × 10^6^ PBMCs or submandibular lymph nodes (LNs)-derived lymphocytes using RNeasy Mini kit and subjected to RT using QuantiTech RT kit (Qiagen, Tokyo, Japan) and random primers. BCR IgG VH-coding cDNAs were amplified using a VH3 specific 5′-primer (5′-TCGTCGGCAGCGTCAGATGTGTATAAGAGACA GAAGGTGTCCAGTGTGARGTGCG-3′), and an IgG-specific 3′-primer (5′-GTCTCGTG GGCTCGGAGATGTGTATAAGAGACAGGCCCTTGGTGGAGGCTGAGGAGACGGT GAC-3′). PCR amplification was performed with a set of unique 8 bp Illumina barcodes to obtain DNA libraries, which were subjected to 2 × 300 bp paired-end indexed sequencing using Illumina MiSeq.

PCR amplicon deep sequencing of BCR VH region was performed by the Illumina MiSeq reagent kit v3 on the MiSeq instrument (Illumina, Tokyo, Japan) according to the manufacturer’s instructions (the methods for 2 × 300-mer paired-end). Sequencing reads were analyzed by Cutadapt to remove the adaptors and Trimmomatic to remove low-quality reads (QV <20) [23,24]. Trimmed paired-end reads (≥50 bp) were joined into a single amplicon sequence using fastq-join [25]. The type of VH gene was predicted by IgBLAST v1.4 with database of V, J, and D genes of rhesus monkeys [26,27]. The top 30 large amplicon sequences in terms of the read counts, which were determined as VH3.33 by IgBLAST, in individual samples were subjected to phylogenetic analysis, except for the samples from macaque #1, in which only five amplicon sequences were subjected to phylogenetic analysis due to the limitation of obtained sequence reads. A total of 124 B404-class VH controls obtained in the previous study [16], and 60 non-VH3.33 sequences derived from #3 were also added. The phylogenetic analysis was performed using MEGA [28]. The short-read sequences obtained by NGS were deposited in the DNA Data Bank of Japan (in submission).

## 3. Results

### 3.1. Germline Ig VH3.33 Polymorphisms in Rhesus and Cynomolgus Macaques

We first examined germline Ig VH3.33 polymorphisms using available PBMCs derived from Burmese rhesus (*n* = 28) and cynomolgus (*n* = 24) macaques (Table 1). In rhesus macaques, polymorphisms at the residue 38, V (valine; 38V) or E (glutamic acid; 38E), and at the residue 65, I (isoleucine; 65I) or T (threonine; 65T), were found. There were two genotypes, VH3.33_VI (38V-65I) and VH3.33_ET (38E-65T), with allele frequencies of 30/56 in VI and 26/56 in ET. Fifteen of twenty-eight rhesus macaques possessed VH3.33_ET allele. In contrast, these polymorphisms were not observed, and only VH3.33_VI was found in cynomolgus macaques.

### 3.2. Neutralizing Antibody Responses in SIVsmE543-3-Infected Rhesus Macaques

We investigated neutralizing antibody induction associated with the Ig VH3.33 polymorphisms by using plasma and PBMC samples obtained from seven SIVsmE543-3-infected rhesus macaques (#1–#7) in our previous study. Four (#4, #5, #6, and #7) of the seven macaques received a DNA-prime/SeV-Gag vaccine [18,19] approximately 3 months before the SIVsmE543-3 challenge. Data on viral loads in macaques #6 and #7 were previously reported [19], but the data on the remaining five macaques have not been published. Analysis of germline Ig VH3.33 polymorphisms revealed that macaques #1 and #7 had no VH3.33_ET, whereas the remaining five macaques possessed VH3.33_ET; macaques #1 and #7 had VH3.33_VI/VI, while macaques #2 and #4 had VH3.33_VI/ET and macaques #3, #5, and #6 had VH3.33_ET/ET (Table 2).

**Figure 1:**
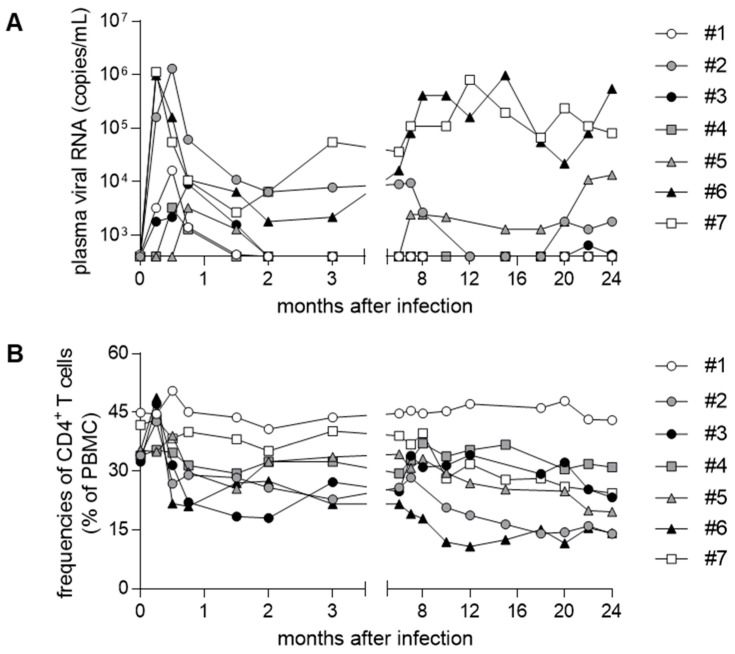
Plasma viral loads and peripheral CD4 frequencies in rhesus macaques after SIVsmE543-3 infection. (**A**). Plasma viral loads (SIVsmE543-3 *gag* RNA copies/mL plasma) were determined as described previously [19]. The lower limit of detection is approximately 4 × 10^2^ copies/mL. (**B**). Peripheral blood CD4^+^ T-cell frequencies. Data on viral loads and CD4^+^ T-cell frequencies in macaques #6 and #7 were previously reported [19], while data on the remaining five macaques have not been published.

We also determined TRIM5 polymorphisms, which are known to have influence on susceptibility to SIVsmE543-3 infection [21]. Five (#1, #2, #3, #4, and #5) of the seven macaques possessed the TRIM5α_TFP allele, which is known to confer resistance to SIVsmE543-3 infection (Table 2). These five macaques with TRIM5α_TFP consisting of three unvaccinated (#1, #2, and #3) and two vaccinated (#4 and #5) showed lower viremia; viremia was undetectable at the setpoint (after two months post-infection) in four of them, while viremia became undetectable after one year post-infection in the remaining one (#2) (Figure 1). Viremia was detected from week 1 post-infection in the three unvaccinated macaques, but detection of viremia was delayed in the two vaccinated macaques (initial detection at week 2 in #4 and at week 3 in #5) (Figure 1). However, it was difficult to find vaccine efficacy as even the unvaccinated macaques controlled viremia, possibly due to TRIMα_TFP-mediated resistance to infection. In contrast, macaques #6 and #7, not possessing TRIM5α_TFP, failed to control SIVsmE543-3 replication with higher viremia as reported previously [19] (Figure 1).

**Figure 2:**
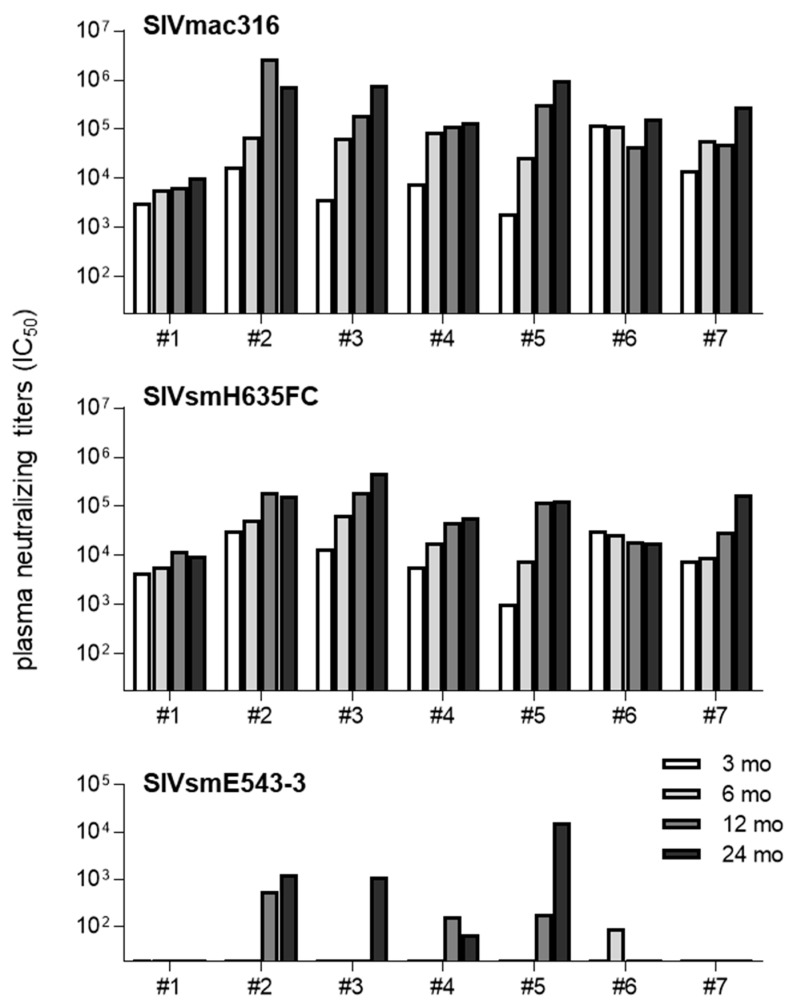
Plasma NAb responses in rhesus macaques after SIVsmE543-3 infection. Plasma neutralizing titers (plasma dilution of half maximal inhibitory concentration (IC_50_)) against SIVmac316 (top), SIVsmH635FC (middle), and SIVsmE543-3 (bottom) at month 3, 6, 12, and 24 post-infection are shown.

We examined post-infection plasma NAb titers against NAb-sensitive SIVmac316 and SIVsmH635FC and NAb-resistant SIVsmE543-3 in the chronic phase of SIVsmE543-3 infection (Figure 2). All macaques showed efficient induction of NAb responses against NAb-sensitive SIVmac316 and SIVsmH635FC at three months post-infection. In contrast, NAb responses against NAb-resistant SIVsmE543-3 were induced only in the five macaques (#2, #3, #4, #5, and #6) possessing VH3.33_ET, mostly one year post-infection. Even macaque #4, without detectable viremia since one month post-infection, showed detectable anti-SIVsmE543-3 NAb responses, while macaque #7, not possessing VH3.33_ET with higher viremia, did not induce detectable anti-SIVsmE543-3 NAb responses. These results suggest anti-SIVsmE543-3 NAb induction associated with VH3.33 polymorphisms in NAb-resistant SIVsmE543-3 infection.

### 3.3. Analysis of BCR VH3.33 Sequences Derived from PBMCs and LNs by NGS

**Figure 3:**
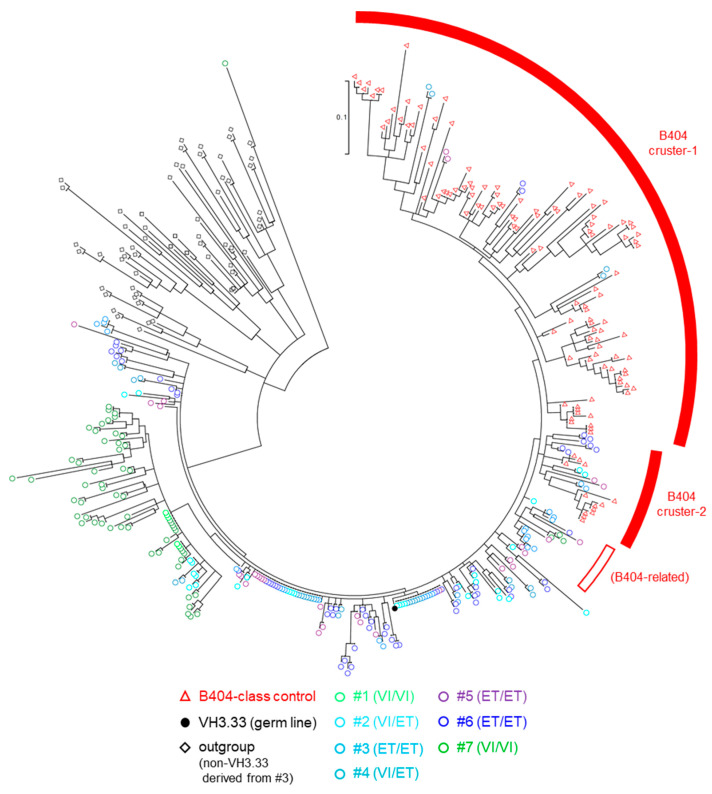
Phylogenic analysis of BCR-VH3.33 cDNA. NGS analysis of IgG VH genes. PBMCs at month 24 of all macaques and LNs at month 24 of macaques #2 and #6 were used. BCR VH regions were amplified from these sample-derived RNAs using a VH3-specific forward primer and an IgG constant region-specific reverse primer. The top thirty large sequences in terms of the read counts, which were determined as VH3.33 by IgBLAST, in individual samples (circles) are subjected to phylogenetic analysis, except for macaque #1 PBMC-derived samples, in which only five subjected were used due to the limitation of obtained sequence read counts. A total of 124 B404-class VH controls obtained in our previous study (red triangles) [16] and 60 non-VH3.33 sequences derived from #3 (black diamonds) were added as controls.

To examine B404-class Ab induction, we performed NGS analysis of BCR VH3.33 genes in the seven macaques. IgG VH cDNAs derived from PBMCs and LNs at month 24 post-infection were amplified by using a VH3-specific forward primer and an IgG-specific reverse primer, and subjected to NGS analysis. Amplicon sequences, which were determined as VH3.33 by IgBLAST and B404-class VH controls obtained in the previous study [16] were subjected to phylogenetic analysis (Figure 3). We determined two B404 clusters (cluster-1 and cluster-2) and some branches beside B404 clusters (B404-related). Sequences derived from macaques possessing VH3.33_ET allele (#2, #3, #4, #5, and #6) but not those derived from macaques without VH3.33_ET (#1 and #7) were included in B404 cluster-1 or cluster-2, suggesting B404-class antibody induction associated with germline VH3.33 polymorphisms.

**Figure 4:**
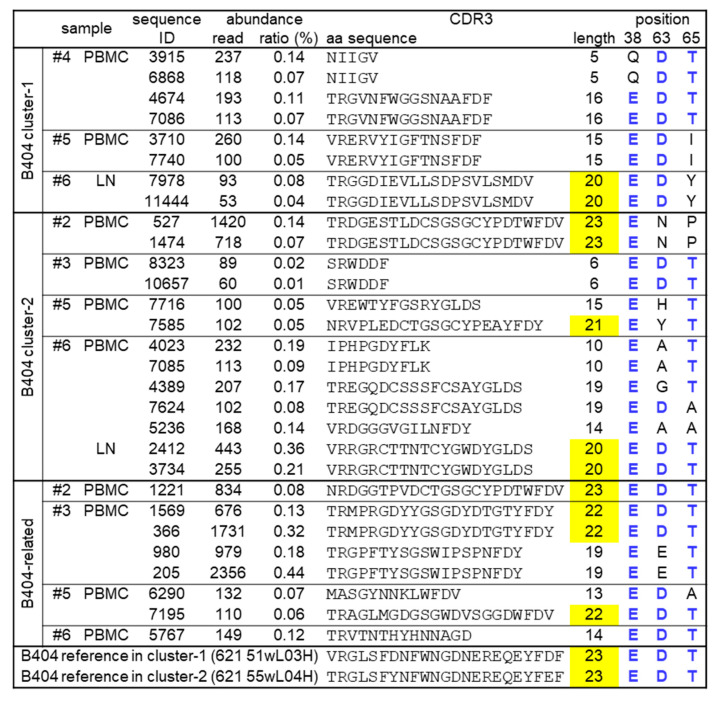
VH sequences clustered with B404-class antibodies. VH sequences more than fifty reads, which were included in B404-class cluster-1, cluster-2, or B404-related, are shown. B404-class VH controls obtained in our previous study are also shown at the bottom [16]. In individual sequences, derived samples, abundance, CDR3 amino acid (aa) sequences and lengths, and the residues 38, 63, and 65 in VH3.33 are shown. The CDR3 lengths with 20 aa or longer are highlighted in yellow. Blue letters mean amino acids which are identical to those in B404-class Abs in our previous study [16].

The B404-class NAbs have heavy chains using VH3.33 with long CDR3 [15]. We found VH3.33 sequences having long CDR3 (20 amino acids length or longer) clustered with B404-class controls derived from macaques #2, #5, and #6 (Figure 4). There were also VH3.33 sequences having long CDR3 derived from macaques #2, #3, and #5 in the B404-related branch. No VH3.33 sequence having long CDR3 was found in macaque #4 that possessed VH3.33_ET but showed no detectable viremia in the chronic phase (after one month post-infection).

## 4. Discussion

Our previous study reported a germline VH3.33 polymorphism-dependent induction of B404-class antibodies that can neutralize NAb-resistant SIVsmE543-3 in NAb-sensitive SIVsmH635FC infection [16]. In the present study, we examined whether even NAb-resistant SIVsmE543-3 infection can elicit B404-class antibody responses. In SIVsmE543-3 infection, macaques possessing VH3.33_ET (#2, #3, #4, #5, and #6) induced NAb responses against not only NAb-sensitive SIVmac316 and SIVsmH635FC, but also NAb-resistant SIVsmE543-3 (Figure 2). In contrast, macaques not possessing VH3.33_ET (#1 and #7) induced NAb responses against only NAb-sensitive SIV, but not SIVsmE543-3 (Figure 2). These results imply anti-SIVsmE543-3 NAb induction associated with germline VH3.33 polymorphism in NAb-resistant SIVsmE543-3 infection. NGS analysis of Ig VH sequences detected VH3.33 sequences clustered with B404-class NAbs only in macaques possessing VH3.33_ET, but not in those without VH3.33_ET (Figure 3). These results indicate B404-class antibody induction associated with germline VH3.33 polymorphisms, even in NAb-resistant SIVsmE543-3 infection.

In our previous study, analysis using germline-reverted B404 mutants revealed that the 38E in VH3.33 is responsible for B404-class antibody binding to SIVsmH635FC Env [16]. A germline-reverted B404 with 38E could also recognize SIVsmE543-3 Env, however it remains unclear whether the potential of NAb-resistant SIVsmE543-3 Env to bind to the origin of B404-class antibody or to prime B cells for B404-class antibody responses is comparable to that of SIVsmH635FC Env. The present study indicates that even NAb-resistant SIVsmE543-3 Env can prime B cells for B404-class NAb induction.

Five macaques possessing TRIM5α_TFP showed lower plasma viral loads, whereas two macaques without TRIM5α_TFP had higher viral loads (Table 2 and Figure 1). Higher viral loads are expected to contribute to higher antibody responses by repeated antigen-BCR interactions. Indeed, anti-SIVmac316 and anti-SIVsmH635FC NAb responses are relatively high at three months post-infection in macaques #6 and #7, possessing no TRIM5α_TFP and having higher viral loads (Figure 2). However, anti-SIVsmE543-3 NAb responses were undetectable in macaque #7 without VH3.33_ET, despite having higher viral loads. In contrast, anti-SIVsmE543-3 NAb responses were induced in all macaques possessing VH3.33_ET and TRIM5α_TFP (#2, #3, #4, and #5) despite having lower viral loads. These results support our conclusion that the germline VH3.33 polymorphism is a determinant for the induction of B404-class antibody responses. The effect of induced anti-SIVsmE543-3 NAb responses on viremia was unclear. In macaque #2, viremia was undetectable after anti-SIVsmE543-3 NAb induction at one year post-infection, whereas macaque #5 maintained viremia even after anti-SIVsmE543-3 NAb induction at one year post-infection.

Transmitted founder viruses in HIV infection are known to be NAb-resistant [29,30]. NAb-resistant HIV Envs have a restricted potential to bind a germline BCR and stimulate Env-specific naïve B cells, resulting in the inefficient induction of potent NAb responses [31,32,33,34,35]. However, our results indicate that the NAb-resistant SIVsmE543-3 Env can bind to the VH3.33_ET-derived BCR and stimulate naïve B cells for NAb induction. Thus, macaques possessing VH3.33_ET could be a useful model to analyze B-cell maturation process to induce potent NAb responses.

In summary, the present study indicates anti-SIVsmE543-3 NAb induction associated with germline VH3.33 polymorphisms in NAb-resistant SIVsmE543-3 infection. Further analysis of B-cell maturation in the process of NAb induction may help us to reveal the mechanism of potent NAb induction.

## Figures and Tables

**Table 1:** Germline Ig VH3.33 polymorphisms in rhesus and cynomolgus macaques ^a^.

Macaque	*n*	VH3.33 Allele ^b^		
		VI/VI	VI/ET	ET/ET
Rhesus	28	13	4	11
Cynomolgus	24	24	0	0

^a^ PBMCs derived from Burmese rhesus macaques used in experiments at TPC/NIBIOHN and cynomolgus macaques at NIID were subjected to analysis of germline Ig VH3.33 polymorphism. ^b^ VI and ET represent VH3.33 alleles with 38V-65I (VH3.33_VI) and 38E-65T (VH3.33_ET), respectively. Numbers of macaques possessing VI/VI, VI/ET, and ET/ET are shown, respectively.

**Table 2:** Burmese rhesus macaques used for analysis of NAb induction after SIVsmE543-3 infection.

Macaques	VH3.33 Allele ^a^		TRIM5 Allele ^b^		Vaccination ^c^
#1	VI	VI	TFP	Cyp	unvaccinated
#2	VI	ET	TFP	Cyp	unvaccinated
#3	ET	ET	TFP	Cyp	unvaccinated
#4	VI	ET	TFP	Cyp	vaccinated
#5	ET	ET	TFP	TFP	vaccinated
#6	ET	ET	Q	Q	vaccinated
#7	VI	VI	Cyp	Cyp	vaccinated

^a^ VI and ET represent VH3.33 alleles with 38V-65I (VH3.33_VI) and 38E-65T (VH3.33_ET), respectively. ^b^ TFP, Q, and Cyp represent TRIM5α_TFP, TRIM5α_Q, and TRIMCyp, respectively. ^c^ Macaques #4, #5, #6, and #7 received a DNA-prime/SeV-Gag vaccine [18,19] approximately 3 months before SIVsmE543-3 challenge.

## Data Availability

The short-read sequences obtained by NGS were deposited in the DNA Data Bank of Japan (in submission).
